# Pedal Power: Explorers and commuters of New York Citi Bikesharing scheme

**DOI:** 10.1371/journal.pone.0232957

**Published:** 2020-06-03

**Authors:** Justine I. Blanford, MGIS Geog 586 Students

**Affiliations:** 1 Dutton e-Education Institute, Pennsylvania State University, State College, PA, United States of America; 2 Department of Geography, Pennsylvania State University, State College, PA, United States of America; University of British Columbia, CANADA

## Abstract

Bike share schemes are increasing in popularity. During 2013, New York City (NYC) launched a bike sharing scheme, Citi Bike, to provide users with the ability to cycle around the city. How these bikes are used is useful for understanding sustainability and infrastructure needs in urban cities. In this study spatial analysis methods were used to analyze space and time usage patterns during a 12 month period. We found that bike usage varied over the months with the lowest number of rentals occurring during the winter months (N = 200,000) and highest during the summer months (N > 1 million trips). Bike use varied spatially and temporally by user type (customer vs subscriber) and gender (male vs female). Over 100,000 unique routes (origin-destinations) were identified with the top five most popular routes starting and ending at the same station location. When comparison of existing bike distributions were made with bike use patterns, supply gaps were identified. The findings are useful for enhancing infrastructure needs and provide a basis for future comparisons to be made as the system changes over time.

## 1.0 Introduction

More than 450 bike sharing systems exist worldwide [1] since their inception nearly 50 years ago [2]. Bike share programs provide a low-cost, short-term and healthy public transport option for cities [3] enabling users to borrow bicycles from automated docking points or stations for a fee. Once users have finished with the bicycle they can return the bicycle to an empty docking station at another stand or location within the geographic area covered by the bike sharing scheme. Many policy makers, health professionals as well as transport and urban planners are seeking to promote and facilitate cycling as a sustainable and healthy mode of transport [4, 5], therefore understanding how this type of transport is used will help in the longevity and success of such transport options.

New York City (NYC) has a number of ground transportation assets that consists of taxis, buses, a subway system, ferries, and now a bike share system. An affordable bike share system, operated by NYC Bike Share LLC was launched in May 2013 throughout Manhattan and Brooklyn [6]. Bikes can be used for quick short trips around NYC [6] and are aimed at users age 16 years or older [7]. Between 2013 and August 2014, there were three options available for renting bikes that included an annual membership ($95 / annum) for rides up to 45 minutes and a 24-hour pass ($9.95) or a 7-day pass ($25) for rides up to 30 minutes [8]. For longer trips an overtime fee was charged depending on the duration of use (S1 Table). Although the bike share system has expanded since its inception, its use during its first year has not been fully analyzed and is the main purpose of this study.

Several studies have analyzed origin—destination and start and end times associated with bike share data to identify usage characteristics [9, 10, 11, 3, 12, 13] and commuting dynamics. For example, [13] found six usage patterns based on temporal characteristics that were useful for demographic and community detection in the data. Several cities exhibited two commuter peaks during weekdays and one peak at the weekend (e.g. Bordeaux, Boston, Changwon, London, Mexico City, Milan, Montreal, Paris, Rennes, Tel Aviv, Toronto and Washington DC), thus capturing commuters and weekend leisure users.

Gender-biases in bike usage have been found in several studies (e.g. [14, 15, 16, 17, 18]). Women are less likely to use bicycles for commuting purposes than men [19, 20, 21, 22, 23, 24]. Although this does vary from location to location (e.g. 1 in 5 bike commuters are women in a study from Australia [25, 26, 27] versus similar usage rates among men and women in the Netherlands, Denmark and Japan [20, 23]), some of the reasons for lower usage rates in women have been attributed to factors that include “risk averse” behaviors [28] such as the preference for cycling in areas with lower traffic speeds or where bike paths are segregated from the main traffic [29]). Other major barriers to cycling include: distances to destinations; time; infrastructure and end-of-trip facilities; level of organization required, and the carrying of bulky or heavy items [20, 30]. Any combination of these factors may shape journeys of riders [29].

Not only have women been found to be less prolific users of bike share programs but usage patterns between men and women also vary both in time and space. For example, [12] showed that in London the top 100 journeys made by men and women varied spatially and temporally. Spatial variation in commuting patterns have also been found when comparing morning and evening peak flows [11]. Thus the purpose of this study was to examine if different users use space differently in NYC through bike share data.

## 2.0 Methods

### 2.1 Data

**Fig 1:**
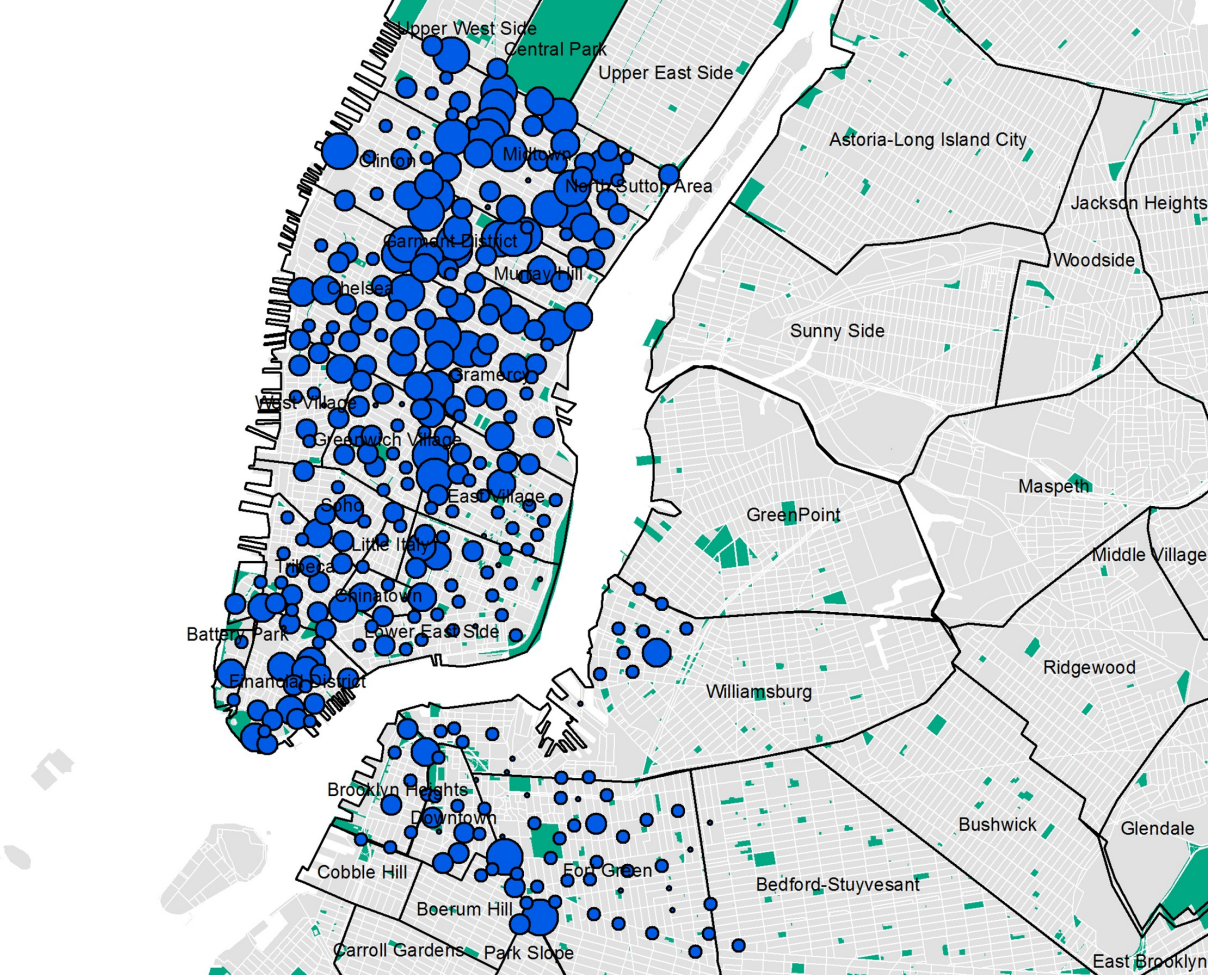
Distribution of bike share docks in NYC. (Data Sources: CitiBike Stations, NYC Parks, Census Blocks, NYC Neighborhood Names (S2 Table)).

New York City (NYC) Citi Bike Share data was obtained for July 2013 through to August 2014 from https://www.citibikenyc.com/system-data (N = 10,407,546). The data includes, date (month, day, year), trip duration, start time, stop time, start station name and id, end station name and id, latitude and longitude of station id, bike id, usertype (e.g. Subscriber = annual member; Customer = 24-hour or 7-day pass user), gender (1 = male; 2 = female) and year of birth. During the first year a total of 332 bike stations were located in NYC; of these 325 were in service and used in this study (Fig 1). Movement of bikes by Citi Bike Staff due to inspection and rebalancing as well as trips that were shorter than 60 seconds in length were removed prior to the data being made available.

Geographic data for New York City was obtained from a variety of sources and used throughout this study to create maps in ArcGIS 10.4 (Environmental Systems Research Institute (ESRI) http://www.esri.com/). All data used in this study are listed in S2 Table.

### 2.2 Analysis

One year of bike usage patterns were examined spatially and temporally (July 2013 to June 2014). First various descriptive statistics were used to capture the breakdown of bike trips by customer type (subscriber vs customer), gender, age, month of year, duration (minutes), popular stations and routes (origin-destination).

The spatial distribution of bike usage and availability of bikes was examined using spatial autocorrelation analysis (Global Moran’s *I* analysis and Local Indicators of Spatial Association (LISA) [31]; [32]) and identify where statistically significant bike use clusters occurred. Analysis was performed using the total number of times a bike was borrowed for a station (O_s,_ (Eq 1)):

$$O_{s}=\sum_{i=1}^{N}S_{i}$$ (1)

Where O_s_ represents the start or end location for a station (*S)*.

A Thiessen polygon containing a single bike station was created, where any location within the polygon will be closer to that bike station than any other bike station. The total number of times a bike was borrowed for each station (O_s,_ Eq 1) was assigned to the polygon representing that unique station. Analyses were conducted to assess bike usage by gender (male vs female), day of the week (weekday (Monday thru Friday) vs weekend (Saturday and Sunday)), user type (subscriber vs customer). These patterns were compared with the distribution of bikes based on the number of bikes available at each station.

A Nearest Neighbour Analysis (NNA) was used to assess the distribution of bike stations, clustering of bikes and the average distance between each station.

Bike routes refer to the origin-destination (OD) of a trip (ODT) and were created by connecting the start station and end station for each unique bicycle. Since we do not know the actual route that a person may have taken, we calculated the straight line distance between the two stations to obtain a value that represents how far stations were located from each other. Similar to above, we assessed the popularity of a route by calculating the total number of times a route was used during the 12 months using Eq 2.

$$ODT=\sum_{i=1}^{N}{OD}_{i}$$ (2)

Where ODT represents the total number of times a route (OD) was used.

We further examined changes in usage patterns at different times of the day that included 4 time periods–morning peak (6am-8am), afternoon peak (4pm-6pm), morning non-peak (9am-3pm) and afternoon non-peak (7pm-5am). Temporal breakdowns were selected based on preliminary analysis of frequency of bike use throughout a 24hr time period. The time periods were identified when a sudden change in frequency occurred. For example, a peak time period occurred when there was a sudden change in usage such as from 5,000 users to 10,000 users or vice versa in an hour. Thus, for each of the 4 time periods we selected routes associated with each of these and calculated the total number of times each route was used during that time using Eq 2. These were then used to capture commuting patterns as well as show different usage patterns throughout a 24hour time period.

Statistical analyses were also conducted to determine the significance of use between user type (customer vs subscriber), gender (male vs female for subscribers) and day of the week (weekday vs weekend). The average time a bike was used and the total number of times a bike was borrowed was summarized for each station (Eq 1). A Kruskall-Wallis test was used to compare between groups and performed in R (version 3.3).

## 3.0 Results

### 3.1 Bike usage

During the first year of the launch of Citi Bikes the total number of annual memberships purchased increased steadily (Table 1). Total number of short-term bike hires varied over the months with the lowest number of rentals occurring during the winter months (N_24-hourpasses_ = 8,800/day (February);N_7-daypasses_ = 9/week (February)) and highest during the summer months (N_24-hourpasses_ = 38,000/day (September);N_7-daypasses_ = 205/week (July)) (Table 1).

**Table 1:** Total number of memberships, 24-hour and 7-day passes purchased between July 2013 and August 2014.

Month	Total No. Memberships (annual subscribers)	No. 24-hour passes	No. 7-day passes	Average No. 24-hour passes per day	Average No. 7-day passes per week
*Jul-13*	1,852,008	62,273	6,346	31,565	205
*Aug-13*	2,236,665	67,097	5,749	37,009	185
*Sep-13*	2,448,471	52,045	4,634	38,731	154
*Oct-13*	2,808,575	34,234	Unavailable	36,639	Unavailable
*Nov-13*	2,828,230	16,139	Unavailable	24,390	Unavailable
*Dec-13*	2,963,188	7,480	Unavailable	15,499	Unavailable
*Jan-14*	2,989,964	2,876	327	10,556	11
*Feb-14*	2,722,308	3,090	244	8,834	9
*Mar-14*	3,064,063	9,651	735	15,420	24
*Apr-14*	3,077,292	25,699	1,880	24,336	63
*May-14*	3,327,665	40,615	3,260	30,806	105
*Jun-14*	3,366,759	43,856	3,658	34,523	122

(Source: Citi Bike https://www.citibikenyc.com/system-data).

**Fig 2:**
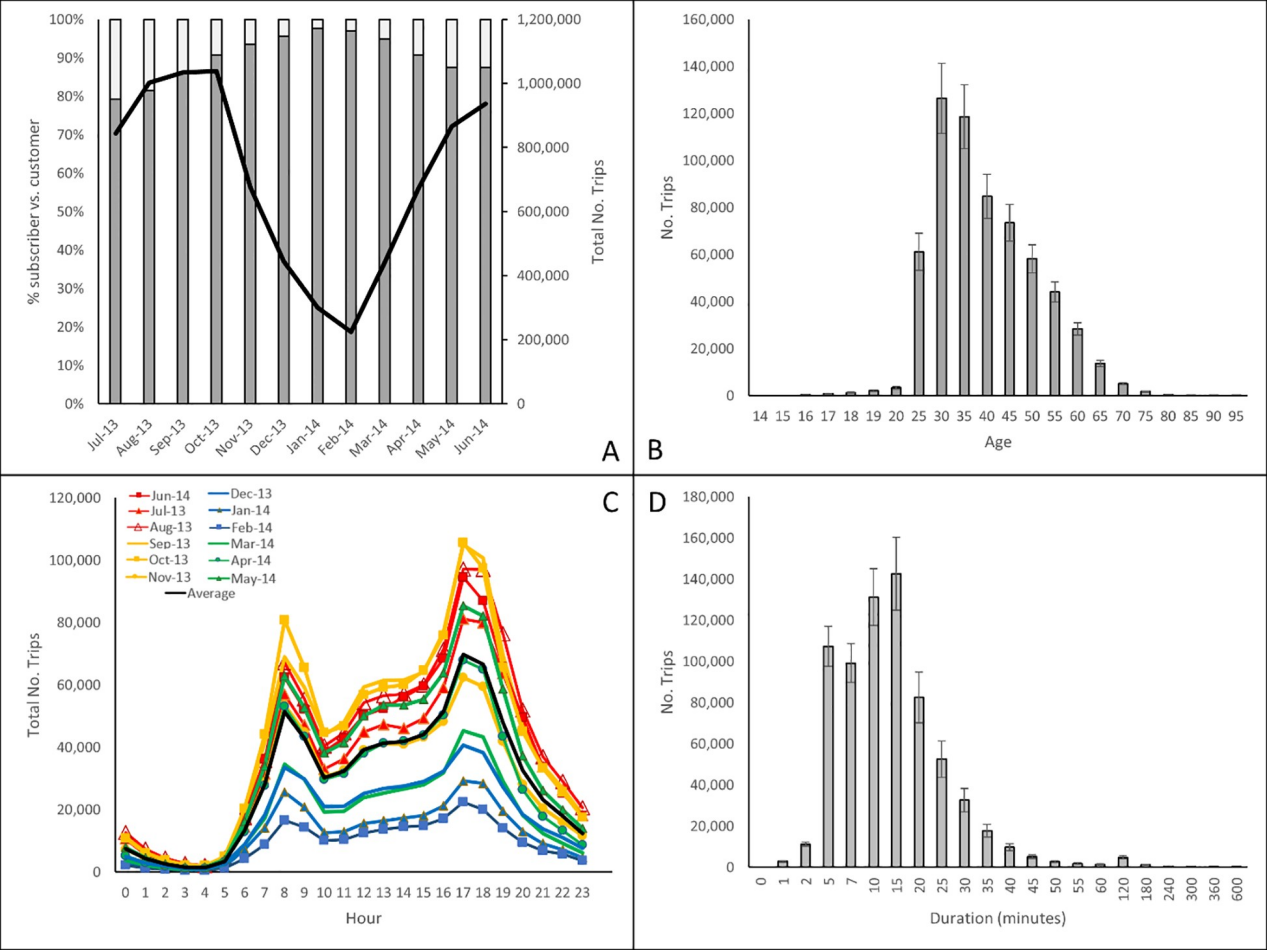
Breakdown of bike trips between July 2013 and June 2014 by **(A)** customer (subscriber (grey bars) vs customer (white bars); total number of trips (solid black line)), **(B)** age (+/-SE), **(C)** time of day (blue = winter months, red = summer months, green = spring months, brown/yellow = autumn months, black line = average) and **(D)** duration (minutes) (+/-SE).

**Fig 3:**
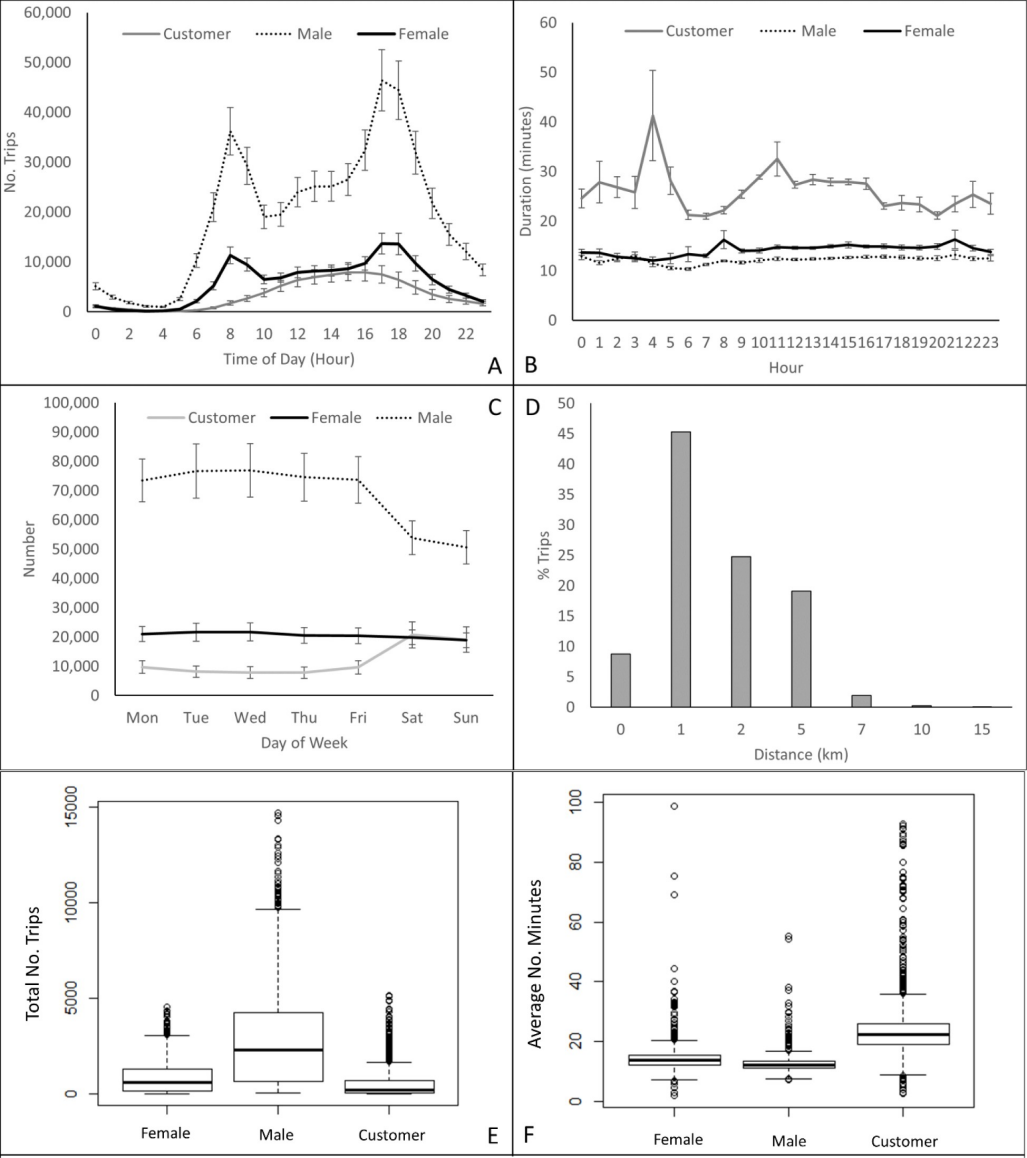
Summary of bike trips by gender (male, female and customer) by **(A)** time of day (+/-SE), **(B)** duration (minutes) (+/-SE), **(C)** day of the week (+/-SE), **(D)** distance, **(E)** number of trips, and **(F)** average duration of trips.

Bike usage in NYC was highest during the summer and autumn months peaking in October 2013 with over 1 million trips recorded. Lowest usage was recorded during the winter months with just over 200,000 trips being made during February, 2014 (Table 2, Fig 2A and 2C). The majority of users were annual subscribers with a maximum of 20% of all trips being made by short-term customers (e.g. 24-hour or 7-day pass customers) (Kruskall-Wallis chi-squared = 412.52, df = 1, p-value < 2.2e-16). The mean age of bike users ranged from 14 to > 80 with the majority of users ranging in age between 30–40 years (Fig 2B). Bike usage peaked twice during the day; once in the morning at 8am and again between 4-6pm with a small increase between 12–2 o’clock (Fig 2C). The majority of trips took between 5–15 minutes (Fig 2D) with variations between months (range 11–16 minutes, Table 2). Bikes were predominantly used by males (mean: 65%; range: 60–78%) in comparison to females (mean: 20%; range: 18–21%) (Table 2, Fig 3A and 3E) (Kruskal-Wallis chi-squared = 246.42, df = 1, p-value < 2.2e-16). The peak usage time between male and female users were consistent (Fig 3A) but usage times varied for customers peaking at 2pm (Fig 3A). Duration of bike use was significantly shorter for male than females; men spent about 10 minutes on the bike while women spent 15 minutes cycling (Kruskal-Wallis chi-squared = 153.37, df = 1, p-value < 2.2e-16). Customers took 20 to 40 minute longer rides than subscribers (Fig 3B and 3F) (Kruskal-Wallis chi-squared = 488.47, df = 1, p-value < 2.2e-16). Bike use dropped significantly on weekends (Kruskal-Wallis chi-squared = 213.88, df = 1, p-value < 2.2e-16) in particular for male users but increased for customer users (Fig 3C). The majority of trips (45%) were about 1km in length (Fig 3D).

**Table 2:** The total number of bike trips between July 2013 and June 2014, the mean number of trips per day (+/-SE), mean durations (+/-SE), gender breakdown and the number of unique routes taken during each month.

	Trips	Total Trips/day	Duration (minutes)	Gender (%)			Unique Routes
Month	Total	Mean (+/-SE)	Mean (+/ SE)	Male	Female	Unknown	Total
*Jul-13*	843,416	27,207(994)	16.64 (0.13)	60.64	18.62	20.74	77,878
*Aug-13*	1,001,958	32,321(829)	16.33 (0.04)	61.57	19.86	18.57	79,143
*Sep-13*	1,034,359	34,479(746)	15.09 (0.03)	64.75	21.21	14.04	78,011
*Oct-13*	1,037,712	33,475(858)	13.77 (0.03)	68.83	21.77	9.40	75,261
*Nov-13*	675,774	22,526(1,437)	12.86 (0.07)	72.12	21.36	6.51	65,054
*Dec-13*	443,966	14,321(1,205)	12.54 (0.12)	75.59	20.04	4.37	55,565
*Jan-14*	300,400	9,690(1,004)	12.24 (0.16)	78.60	18.98	2.42	47,230
*Feb-14*	224,736	8,026(746)	14.58 (0.19)	78.55	18.46	3.00	43,000
*Mar-14*	439,117	14,165(790)	11.93 (0.02)	75.28	19.50	5.22	56,708
*Apr-14*	670,780	22,359(1,114)	13.87 (0.02)	70.57	20.02	9.41	64,997
*May-14*	866,117	27,939(937)	14.67 (0.02)	67.39	20.18	12.42	70,484
*Jun-14*	936,880	31,229(1,300)	14.88 (0.02)	66.28	21.09	12.63	72,175

### 3.2 Density of bikes

**Fig 4:**
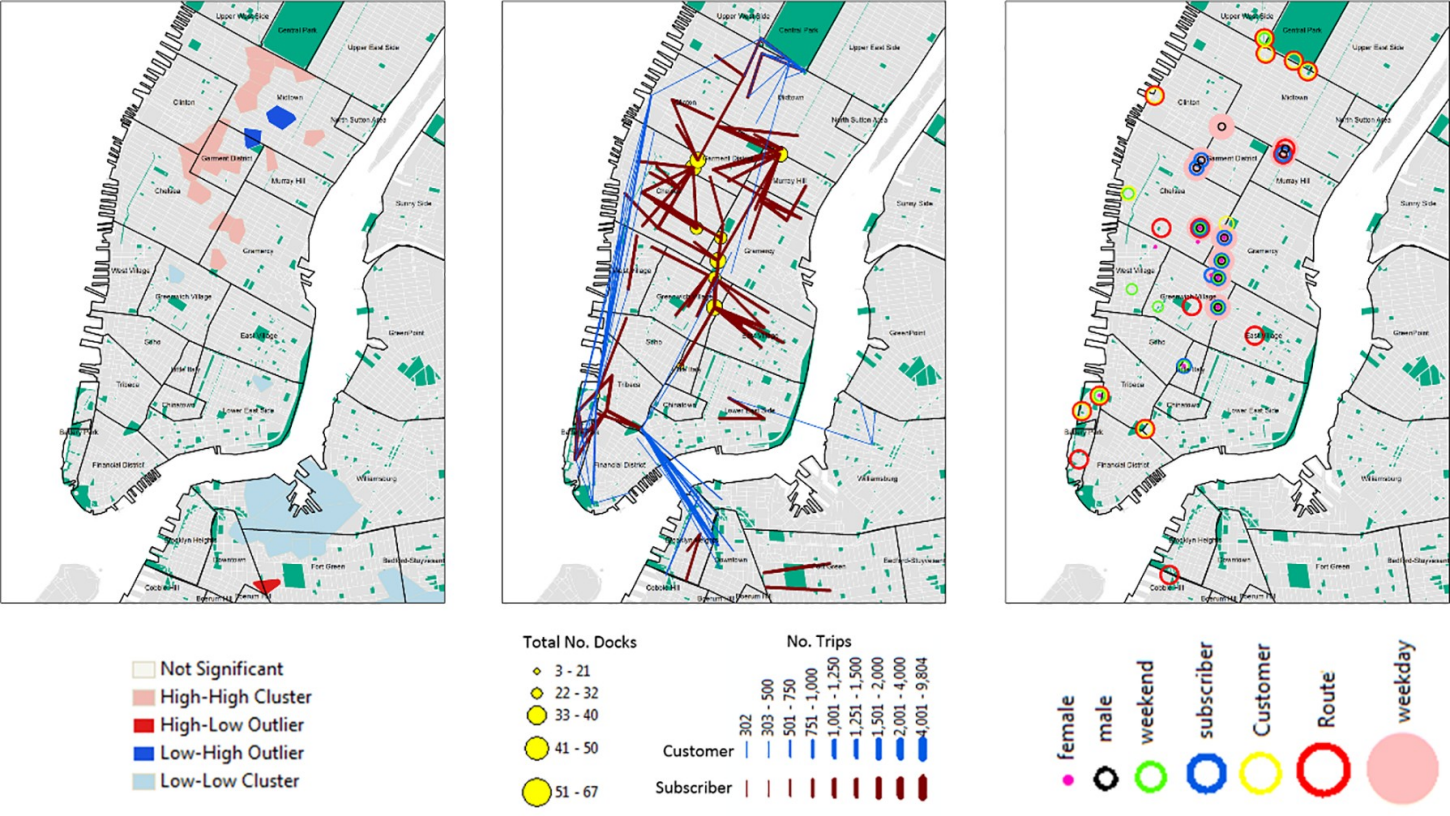
**(A)** Distribution of bike stations and clustering of bikes (based on total number of docks at each bike station); **(B)** most popular start locations (showing the top 10 start stations) and origin-destinations (top 100 routes used by subscribers and customers); and **(C)** top ten start stations by gender (male and female), user type (subscriber and customer), day of the week (weekend (Saturday and Sunday) and weekday (Monday through Friday)) and route. (Data Sources: CitiBike Stations, NYC Parks, Census Blocks, NYC Neighborhood Names (S2 Table)).

Although bike stations were dispersed (NNA = 1.06; Z-score = 2.14; p = 0.032) with a mean observed distance of 0.22 km throughout lower Manhattan extending into parts of Brooklyn (Figs 1 and 4A), bikes were clustered (Moran’s I = 0.31,Z-score = 9.44; p = 0.0000) with the highest density of bikes found in the central north of Manhattan (Fig 4A).

The most popular start locations are illustrated in Fig 4B; the top four starting stations had over 100,000 trips recorded and include E42 St & Vanderbilt Ave (519), 8 Ave & W 31 St (521), E 17 St & Broadway (497), Lafayette St & E 8 St (293) (Table 3, Fig 4B). The most popular end stations were similar to the start stations (Table 3).

**Table 3:** Top ten ranked start station, end station and origin-destination routes based on the total number of trips recorded between July 2013 and June 2014.

Rank	Start Station	End Station	Route (same start and end location)	Route
1	519; E 42 St & Vanderbilt Ave	497; E 17 St & Broadway	2006_2006; Central Park	318_477; E 43 St & Vanderbilt Ave: W 41 St & 8 Ave
	**No Trips:** 113,001	**No Trips:** 111,696	**No Trips:** 12,407	**No Trips:** 3,259
2	521; 8 Ave & W 31 St	293; Lafayette St & E 8 St	281_281; Grand Army Plaza & Central Park S	363_327; West Thames St: Vesey Pl & River Terrace
	**No Trips:** 110,401	**No Trips:** 100,993	**No Trips:** 5,438	**No Trips:** 3,238
3	497; E 17 St & Broadway	521; 8 Ave & W 31 St	499_499; Broadway & W 60 St	281_499; Grand Army Plaza & Central Park S: Broadway & W 60 St
	**No Trips:** 103,807	**No Trips:** 99,199	**No Trips:** 5,271	**No Trips:** 3,141
4	293 Lafayette St & E 8 St	519; E 42 St & Vanderbilt Ave	426_426; West St & Chambers St	116_521; W 17 St & 8 Ave: 8 Ave & W 31 St
	**No Trips:** 103,707	**No Trips:** 91,088	**No Trips:** 3,572	**No Trips:** 2,881
5	435; W 21 St & 6 Ave	435; W 21 St & 6 Ave	387_387; Centre St & Chambers St	294_382; Washington Square E: University Pl & E 14 St
	**No Trips:** 85,874	**No Trips:** 86,672	**No Trips:** 3,331	**No Trips:** 2,875
6	285; Broadway & E 14 St	426; West St & Chambers St	457_457; Broadway & W 58 St	432_293; E 7 St & Avenue A: Lafayette St & E 8 St
	**No Trips:** 85,171	**No Trips:** 85,123	**No Trips:** 2,990	**No Trips:** 2,812
7	426; West St & Chambers St	285; Broadway & E 14 St	514_514; Ave & W 40 St	435_462; W 21 St & 6 Ave: W 22 St & 10 Ave
	**No Trips:** 84,485	**No Trips:** 82,978	**No Trips:** 2,466	**No Trips:** 2,807
8	151; Cleveland Pl & Spring St	382; University Pl & E 14 St	327_327; River Terrace	519_492; E 42 St & Vanderbilt Ave: W 33 St & 7 Ave
	**No Trips:** 77,061	**No Trips:** 78,055	**No Trips:** 2,246	**No Trips:** 2,631
9	490; 8 Ave & W 33 St	151; Cleveland Pl & Spring St	398_398	327_363; Vesey Pl & River Terrace: West Thames St
	**No Trips:** 76,920	**No Trips:** 77,398	**No Trips:** 1,944	**No Trips:** 2,619
10	402; Broadway & E 22 St	402; Broadway & E 22 St	363_363; West Thames St	514_426; 12 Ave & W 40 St: West St & Chambers St
	**No Trips:** 76,409	**No Trips:** 77,147	**No Trips:** 1,766	**No Trips:** 2,577

### 3.3 Bike routes

A total of 101,460 unique routes (origin-destinations) were identified with a minimum of 43,000 different routes used during February and the largest number of different routes recorded during August (N = 79,143) (Table 2). The highest number of trips took place during August and September of 2013 with over 1 million journeys recorded. The top 5 most popular routes originated and finished at the same station location (Table 3) and include several stations around Central Park (N = 12,407), Grand Army Plaza & Central Park (N = 5,438), Broadway & W 60 St (N = 5,271), West St & Chambers St (N = 3,572), Centre St & Chambers St (N = 3,331) (Fig 4B and 4C; Table 3).

**Fig 5:**
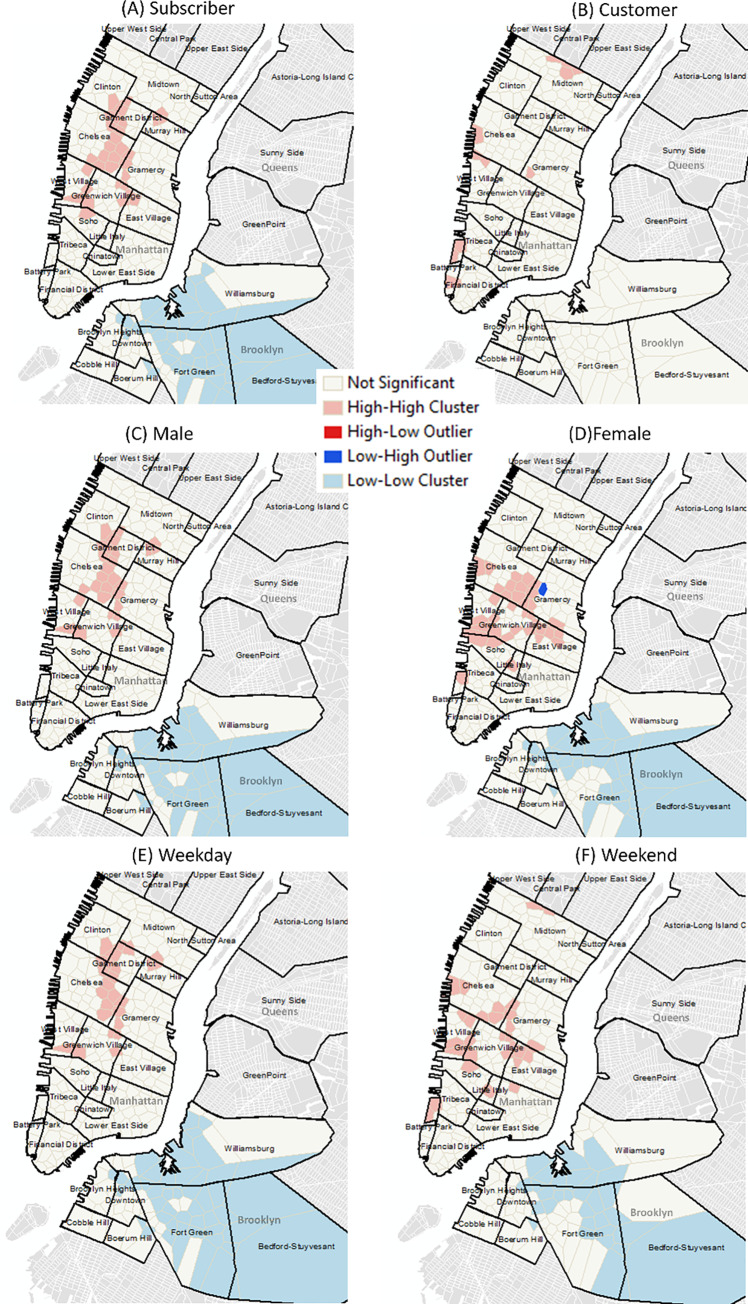
Usage patterns by user type (A) subscribers and (B) customers, (C) male and (D) females as well as during the (E) weekday and (F) weekend. (Data Sources: Census Blocks, NYC Neighborhood Names (S2 Table)).

**Fig 6:**
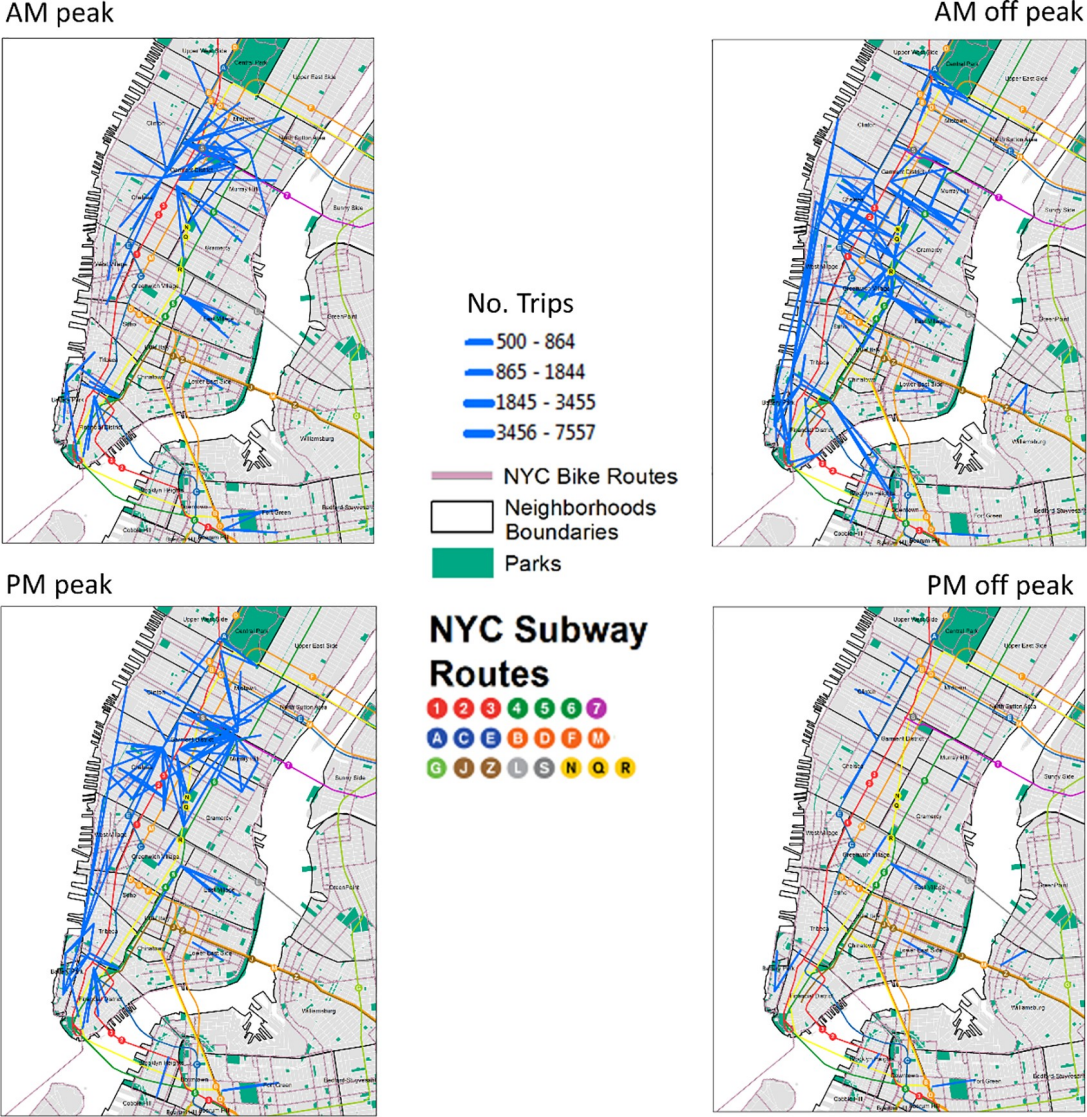
**CitiBike usage at different times of the day (peak morning (AM peak) and afternoon (PM peak); non-peak morning (AM off peak) and afternoon (PM off peak)).** Maps show routes with more than 500 trips. (Data Sources: NYC Parks, Census Blocks, NYC Neighborhood Names, Subway Entrances and Routes, NYC Bike Routes (S2 Table)).

Cluster analysis of bike usage clearly highlighted different use patterns by user type, gender and day of the week (Figs 4C, 5A and 4D). Subscribers were the highest users and clearly used the bikes in the central areas of Manhattan while customers were less abundant and more likely to use areas along the coast and in and around central park (Figs 4B, 5A & 5B). Patterns of male usage were also different from those of females (Fig 5C & 5D) and closely matched those found during the week and weekend (Fig 5E & 5F). Further variations in patterns were also found when bike route use was analyzed at different times of the day (Fig 6).

## 4.0 Discussion/Conclusion

New York City is relatively flat with a favorable climate that is suitable for cycling [33]. Every day trips are made by New Yorkers, many of which are short enough to be covered by bike [34] with the majority of users traveling between 5–15 minutes between locations. Although there are many factors that deter cycling in large cities (e.g. traffic, pollution, poor street conditions, inadequate bike parking and prolific bike theft [33]), in the past decade many cities have improved rider safety by expanding bike networks through the establishment of clearly marked bike lanes (e.g. NYC since 2000 [33]). As a result of improving infrastructure, many cities now have bike share programs, with rising memberships, and are thus providing “healthier” and “friendly” cities through urban re-engagement and the provision of environmentally sustainable travel [35].

Similar to other studies we found distinctive bike usage patterns at different times of the day (peak vs non-peak morning and afternoon) [11, 13], weekdays (weekday vs weekend) as well as between different users (subscribers vs customers; male vs female) [11, 3, 12] (Figs 4B,4C and 5A–5F). Two strong peaks were identified for subscribers, one in the morning and one in the evening suggesting that the bikes were predominantly used for commuting. This remained true for both male and female subscribers. When analyzed by day of the week a drop in demand occurred during the weekend. Distinctive commuting patterns were also highly noticeable throughout the day varying between morning and afternoon peaks and off peak times (Fig 6). Customers, on the other hand, had a single peak (12-4pm) and spent a longer duration on the bikes (than subscribers) suggesting that these users mainly used bikes for leisure activities going longer distances (Fig 4B).

Citi Bikes were predominantly used by males (65–75%) with 20% of the bikes being used by females. These findings are similar to other studies that highlight gender-biases in bike usage (e.g. [14, 15, 16, 17, 18]). Although women are less likely to use bicycles for commuting purposes than men [19, 20, 21, 22, 23, 24] in NYC, we did find that when women used bikes they did use them for commuting purposes as shown by the two peaks (Fig 3A). We also found that spatial usage patterns differed between men and women (Fig 5C and 5D). Examining the most popular start stations, we found some overlap between stations popular between men and women, but there are clearly some stations that are used more by women (Fig 4C). Understanding reasons associated with the low usage rates as well as differences in spatial usage patterns warrants further investigation to determine whether these are associated with “risk averse” behaviors [28] (e.g. cycling in lower traffic speed areas or where bike paths are segregated from main traffic [12]) or due to distances to destinations; time; infrastructure and end-of-trip facilities; or the need for carrying bulky or heavy items [20, 29].

Demand for bikes were highest during the Summer and Autumn months (Fig 2A & 2C; Table 2) and lowest during January and February and are likely due to weather which has been shown to affect cycling demands [36, 37]. Future work will examine how weather such as rain, snow and temperature affects demand throughout the year.

**Fig 7:**
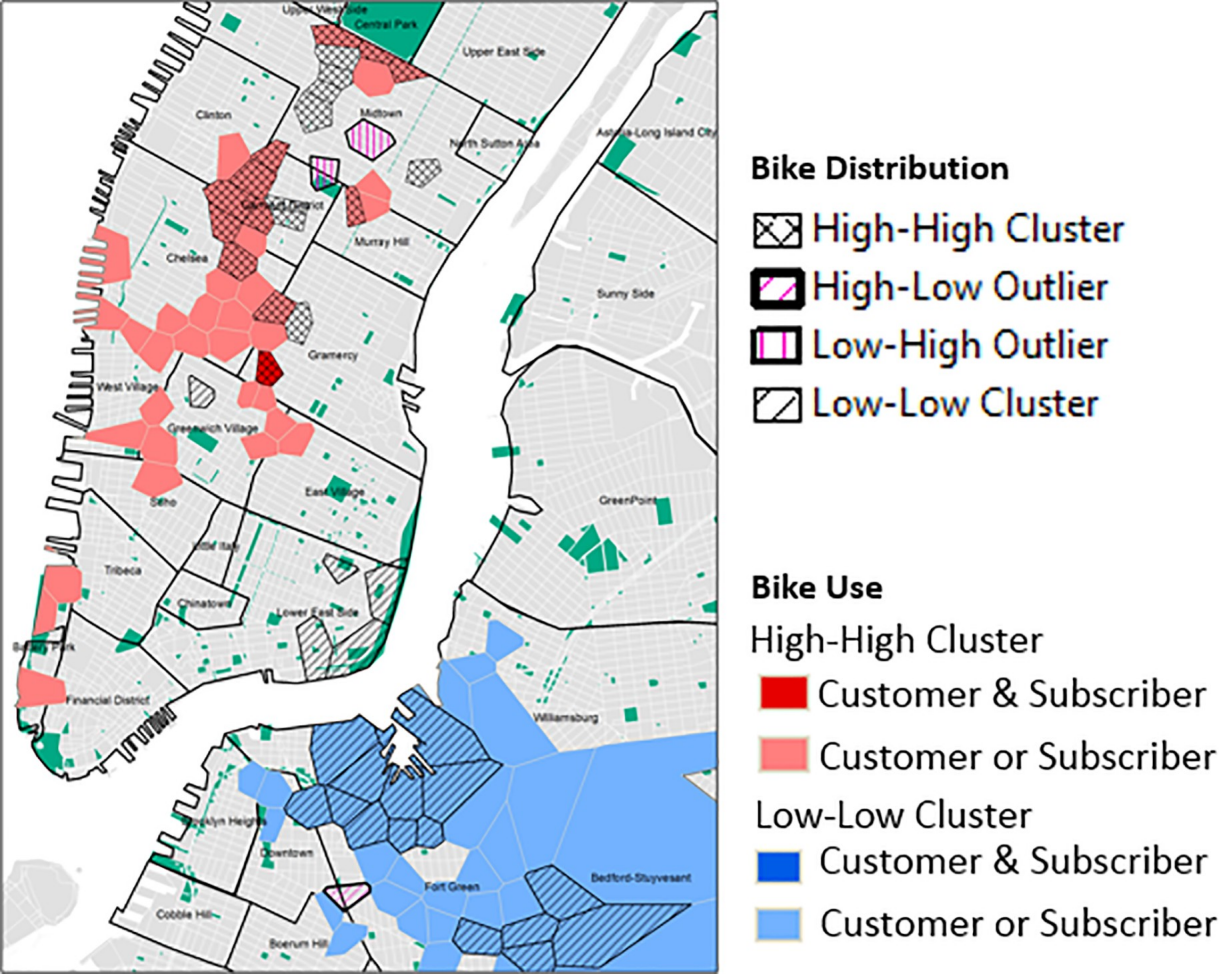
Overlap of statistically significant clusters of the number of bikes with bike usage. (Data Sources: NYC Parks, Census Blocks, NYC Neighborhood Names (S2 Table)).

The origin and destination information obtained through the bike share data were useful for determining movement flows and usage patterns of different user types in NYC and clearly highlighted popular locations and routes. The top 5 most popular routes started and stopped at the same station place suggesting that the bikes were used for leisure activities. This was particularly evident in and around Central Park where the most popular station recorded in excess of 12,000 trips (Fig 4B highlights the 100 most popular routes and Fig 4C start locations). When we compared the distribution of statistically significant high usage stations with the distribution of bikes it became apparent that there was a mismatch in areas of high demand and the availability of bikes (Fig 7). Stations with the highest demand (high-high clusters of subscribers and/or customers) do not necessarily have the highest number of bikes available (high-high clusters) in and around those locations. From our initial investigations further analysis is required to best identify how to redistribute the bike docks in and around the Chelsea/Greenwich Village/Gramercy area and West Village/Soho areas, in particular stations at W 18 St & 6 Ave, W 21 St & 6 Ave, W 15 St & 7 Ave, W 20 St & 7 Ave, Greenwich Ave & 8 Ave, Carmine St & 6 Ave and Barrow St & Hudson St should be considered.

The work presented here shows that bikes are used differently throughout the city by different users. Our study shows a comprehensive overview of usage patterns based on the initial 12 months of the NYC Citi Bike scheme. Knowing key origin-destinations are useful for identifying areas where bike path infrastructure and maintenance should be concentrated to ensure continued safety of riders. For example, popular routes in and around Central Park as well as the bike routes along the Hudson River Greenway and Brooklyn Bridge. The work here provides a foundation that will enable us to examine how use patterns vary over time as costs change and the bike share system expands.

## Supporting information

S1 Table (DOCX)

S2 Table (DOCX)
