# Supplementary material for: Pedal Power: Explorers and commuters of New York Citi Bikesharing scheme
Source: PLoS One. 2020 Jun 3;15(6):e0232957. doi: 10.1371/journal.pone.0232957 (PMC7269338; doi:10.1371/journal.pone.0232957)
Supplement: S1 Table — (DOCX) [file pone.0232957.s001.docx]

**Supp Table 1**: Overtime fees charged for annual membership and 24-hour or 7-day passes ([NYC_CitiBike 2013](#_ENREF_1)).

| Annual Membership (minutes) | Overtime Fees ($) | 24-hour or 7-day Pass (minutes) | Overtime Fees ($) |
| --- | --- | --- | --- |
| <= 45 | 0 | <= 30 | 0 |
| 45-75 | 2.5 | 30-60 | 4 |
| 75-105 | 9 | 60-90 | 13 |
| Every additional 30 minutes | 9 | Every additional 30 minutes | 12 |
