# Supplementary material for: Pedal Power: Explorers and commuters of New York Citi Bikesharing scheme
Source: PLoS One. 2020 Jun 3;15(6):e0232957. doi: 10.1371/journal.pone.0232957 (PMC7269338; doi:10.1371/journal.pone.0232957)
Supplement: S2 Table — (DOCX) [file pone.0232957.s002.docx]

**Supp Table 2:** Summary of GIS data sources used throughout this study.

| Data | Description and Source | URL |
| --- | --- | --- |
| Citi Bike Stations | Citi Bike NYC Station Feed | <http://www.citibikenyc.com/stations/json> |
| NYC Bike Routes | Bike routes. NYC DOT (Department of Transport) | <http://www.nyc.gov/html/dot/html/about/datafeeds.shtml> |
| Subway Entrances and Routes | Subway Entrances  Subway lines | <https://nycopendata.socrata.com/Transportation/Subway-Entrances/drex-xx56>  <http://spatialityblog.com/2010/07/20/more-mta-data-in-gis-format/>  **CUNY Mapping Service at the Center for Urban Research** <http://spatialityblog.com/2010/07/08/mta-gis-data-update/>  <http://spatialityblog.com/2010/05/06/mta-data-in-gis-format/> |
| *Ferry Terminals* | DOITT | <https://data.cityofnewyork.us/Transportation/Ferry-Terminal-Locations/9a56-tyfg> |
| *NYC Neighborhoods* | Neighborhood Boundaries for New York | <http://www.zillow.com/howto/api/neighborhood-boundaries.htm> |
| NYC GIS Zoning Features | New York City Department of City Planning | <http://www.nyc.gov/html/dcp/html/bytes/dwnzdata.shtml>  <http://www.nyc.gov/html/dcp/html/bytes/applbyte.shtml> |
| *NYC Neighborhood Names* | New York City Department of City Planning | <http://www.nyc.gov/html/dcp/pdf/bytes/meta_nhood.pdf>  <http://www.nyc.gov/html/dcp/html/bytes/applbyte.shtml> |
|  |  |  |
| NYC Parks | New York City Department of Parks and Recreation | <https://data.cityofnewyork.us/City-Government/Parks-Properties/rjaj-zgq7> |
| *Railroad Stations* | Railroad passenger stations. UTM NAD 83 Zone 18. Copyright 2001 by NYS Dept of Transportation. | <http://gis.ny.gov/gisdata/inventories/details.cfm?DSID=1263> |
| *Railroad Lines* | Line shapefile of active railroad lines. Copyright 2001 by NYS Dept of Transportation. | <http://gis.ny.gov/gisdata/inventories/details.cfm?DSID=904>  <https://www.dot.ny.gov/divisions/engineering/applications/traffic-data-viewer> |
| *Roads* | Line shapefile showing traffic volumes on significant roads in New York State. NYS Dept. of Transportation (DOT) | <http://gis.ny.gov/gisdata/inventories/details.cfm?DSID=1282> |

NYC_CitiBike. 2013. Pricing. <http://www.citibikenyc.com/pricing> Oct 31, 2014.
